# Forecasting Sauter mean droplet size and examining the range of droplet sizes in a Tenova liquid–liquid extraction column

**DOI:** 10.1038/s41598-024-52542-1

**Published:** 2024-02-08

**Authors:** Neshat Rahimpour, Hossein Bahmanyar, Alireza Hemmati, Mehdi Asadollahzadeh

**Affiliations:** 1 Surface Phenomena and Liquid–Liquid Extraction Research Lab, School of Chemical Engineering, College of Engineering, University of Tehran, Tehran, Iran; 2 School of Chemical Petroleum and Gas Engineering, Iran University of Science and Technology (IUST), P.O. Box: 16765-163, Tehran, Iran; 3 Nuclear Fuel Cycle Research School, Nuclear Science and Technology Research Institute, P.O. Box 11365-8486, Tehran, Iran

**Keywords:** Engineering, Chemical engineering

## Abstract

A new type of Tenova pulsed extraction column was introduced in 2017. It is the newest generation of pulsed columns. Due to the internal equipment of this column and the lack of moving parts and the simplicity and speed of repairs and maintenance, it has been the focus of researchers in recent years. No correlations for predicting the mean drop size and drop size distribution of the Tenova column have been reported. The Sauter mean drop diameter and drop size distribution are investigated for a Tenova pulsed column with a diameter and an active height of 7.4 and 73 cm, respectively. Three standard chemical systems of isobutyl acetate-water, isobutanol-water, and toluene-water have been used. The effects of pulse intensity, dispersed and continuous phase flow rates have been taken into account. In each experiment, 200–300 drops have been analyzed in a total of 10,000 drops. The investigation covered a spectrum of physical properties, notably surface tension (within a range of 1.75–36 mN/m). Operating conditions including pulse intensity (in the range of 0.2–2 cm/s) and the flow rate of continuous and dispersed phases (in the range of 8–30 L/h) have been investigated. Methods based on artificial intelligence (AI) such as multilayer perceptron neural networks and gene expression programming were combined with a dimensional analysis approach to provide a new approach to estimating the mean drop diameter (d_32_). Experimental results have been compared with the equations found by other researchers in similar columns. The variation of drop size distribution has also been experimentally obtained.Methods based on artificial intelligence (AI) such as multilayer perceptron neural networks and gene expression programming were combined with a dimensional analysis approach to provide a new approach to estimating the mean drop diameter (d_32_). Experimental results have been compared with the equations found by other researchers in similar columns. The variation of drop size distribution has also been experimentally obtained.

## Introduction

Liquid–liquid extraction is one of the most widely used separation processes^1,2^. This method is the most common separation process in nuclear and hydrometallurgy industries and the production of relatively pure chemical compounds and environmental waste treatment^3–8^. Pulsed extraction columns are efficient liquid–liquid contactors that have been designed and studied in different types of internal equipment such as packed, tray, and disc and doughnut. One of the advantages of pulsed columns is the absence of moving parts and simplicity and the speed of repairs and maintenance of such columns, while the high efficiency of such columns has caused special attention to such columns^9–14^. The new generation of disc and doughnut columns has been introduced as “Tenova column”. Tenova’s internal equipment has new contact factors on disc and doughnut plate alignments and optimized spacing between plates to achieve less reverse mixing, higher column dispersed phase inventory, and improved mass transfer with a uniform flow rate during operation. The design of an extraction column requires the examination of the separation performance and the detailed examination of the hydrodynamic parameters such as the drop size and the amount of the dispersed phase as well as the flooding velocities to determine the column capacity.

The aim of this study was to investigate the hydrodynamic behavior of Tenva pulse columns and achieve changes in the Sauter mean drop diameter and drop size distribution along the column as the basis for the design of operational units. Experiments were performed along the column using three different liquid–liquid systems.

We incorporated this information to highlight the novelty of the Tenova column and the absence of predictive relationships for crucial parameters, specifically, the average diameter and distribution of drops essential in designing these columns. This article delves into the methodology surrounding alterations in drop size and distribution along the column, elucidating their significance. In addition, methods based on artificial intelligence (AI) such as multilayer perceptron neural networks and gene expression programming were combined with a dimensional analysis approach to provide a new approach to estimating the Sauter mean drop diameter (d_32_). In order to evaluate the proposed model, the data were obtained from the laboratory analysis.

## Previous work

For all extraction columns, drop size is very important in hydrodynamic behavior and mass transfer performance. In other words, the size of the drops affects the presence and residence time of the dispersed phase, it affects the overflow and the interphase surface for mass transfer, and it affects the kinetics of mass transfer due to the mass transfer coefficients that are dependent on the drop diameter. In all extractors, the drop size distribution is not uniform along the length of the column, and the Sauter mean drop diameter is used as the average diameter of diameter size distribution index, which is determined by the relative rate of drop breakage and coalescence and is defined as follows:

1 $$ d_{32} = \left[ {\frac{{\sum\limits_{i = 1}^{n} {{\text{n}}_{{\text{i}}} {\text{d}}_{{\text{i}}}^{3} } }}{{\sum\limits_{i = 1}^{n} {{\text{n}}_{{\text{i}}} {\text{d}}_{{\text{i}}}^{2} } }}} \right] $$

In pulsed columns, the dispersed phase is usually dispersed by a distributor, and usually, the drops have an average diameter between two and five millimeters (two for systems with very low interphase tension and five for systems with high interphase tension).

Kolmogorov^15,16^ predicted the average drop size in pulsed sieve-plate extraction columns based on the theory of isotropic turbulence, which related the drop size to the input mechanical energy.

Hinze^17^ and Shinnar and Church^18^ modified the theory of isentropic turbulence by means of an equation with the surface energy produced by the breaking of drops and the kinetic energy caused by the fluctuations of the same drop volume in the vortex on each side.

2 $$ d_{32} = c_{1} \left( {\frac{\gamma }{{\rho_{c} }}} \right)^{ - 0.6} \psi^{ - 0.4} $$

where the power loss per unit mass of liquids (ψ) can be calculated by the equation proposed by Jealous and Johnson^19^:

3 $$ \psi = \left( {\frac{{\left( {\frac{\pi }{2H}} \right)\left( {1 - \varepsilon^{2} } \right)\left( {Af} \right)^{3} }}{{\varepsilon^{2} C_{0}^{2} }}} \right) $$

For the orifice coefficient (C_0_), a constant value of C_0_ = 0.6 is recommended.

The proposed equations for determining the average diameter of drops based on the Kolmogorov theory describe the effect of pulsation intensity on the size of the drops and predict the diameter of the drops in the range of the mixer-settler regime. Literature missing about the breaking of drops in pulsed sieve-plate extraction columns show that the breaking of drops when passing through the holes of the mesh tray is caused by their shear stress. Anyway, the theory of isotropic turbulence is not suitable for describing the process of drop breaking in mesh tray columns^20^.

Use On the one hand, new physical models have been proposed to predict drop size in pulsed sieve-plate extraction columns. Pietzsch and Pilhofer to model the stability of the drop when passing through the sieve-plate by equating the drag, buoyancy and inertia forces (which cause the drop to break) with the interphase tension force (which causes the drop to remain stable) gave a relation to have achieved^21^:

4 $$ d_{p} = { }\sqrt {\frac{6\sigma }{{\Delta \rho g + { }b\rho_{d} }} + { }\frac{9}{64}{ }\left( {\frac{{c_{D} V_{m}^{2} { }\rho_{c} }}{{\Delta \rho g + { }b\rho_{d} }}} \right)^{2} } - { }\frac{3}{8}{ }\frac{{c_{D} V_{m}^{2} { }\rho_{c} }}{{\Delta \rho g + { }b\rho_{d} }} $$

A summary of other correlations to predict the Sauter mean drop diameter in pulsed columns is given in the Table 1.

**Table 1:** A summary of some predicted correlations of the Sauter mean drop diameter for pulsed columns.

Column type	System	Correlation	References
pulsed sieve plate	-	$d_{32} = 0.92\frac{{(Af)^{ - 0.3} \sigma^{0.5} \mu_{c}^{0.1} }}{{\rho_{c}^{0.6} g^{0.4} }}$	^22^
pulsed sieve plate	Toluene-water	$d_{32} = C\left( {\frac{\sigma }{{\rho_{c} }}} \right)^{0.4} \varepsilon^{ - 0.48} d_{o}^{0.26} h_{p}^{0.34} (Af)^{ - 0.8}$	^23^
Pulse and karr	Different chemical systems	$\frac{{d_{32} }}{{h_{c} }} = \frac{{C\psi \varepsilon^{0.32} }}{{\frac{1}{{1.55\left( {\frac{\sigma }{{{\Delta }\rho gh_{c}^{2} }}} \right)^{1/2} }} + \frac{1}{{0.42\left[ {\left( {\frac{\psi }{g}} \right)\left( {\frac{{{\Delta }\rho }}{g\sigma }} \right)^{1/4} } \right]^{ - 0.35} \left[ {h_{c} \left( {\frac{\Delta \rho g}{\sigma }} \right)^{\frac{1}{2}} } \right]^{ - 1.15} }}}}$	^24^
PDDC	aqueous feed containing ammonium sulphate-Toluene	$$\begin{gathered} \frac{{d_{32} }}{{\sqrt {\frac{\sigma }{\Delta \rho g}} }} = C_{1} \varepsilon^{{n_{1} }} \left( {h\frac{{\sqrt {\rho_{ * } g} }}{{\sigma_{ * } }}} \right)^{{n_{2} }} \left( {\frac{{\mu_{d} g^{1/4} }}{{\rho_{ * }^{1/4} \sigma_{ * }^{3/4} }}} \right)^{{n_{3} }} \left( {\frac{\sigma }{{\sigma_{ * } }}} \right)^{{n_{4} }} \hfill \\ \quad \quad \quad \quad \; \times \left[ {C_{2} + \exp \left\{ {C_{3} \frac{Af}{{\varepsilon \left( {\frac{{\sigma_{ * } g}}{{\rho_{ * } }}} \right)^{1/4} }}} \right\}} \right] \hfill \\ \end{gathered}$$	^25^
PDDC	Kerosene Toluene n-butyl acetate water	$$\begin{gathered} \frac{{d_{32} }}{{\sqrt {\frac{\sigma }{\Delta \rho g}} }} = 33.35 \times 10^{ - 3} \left( {\frac{{Af^{4} \rho_{c} }}{g\sigma }} \right)^{ - 0.283} \left( {\frac{{d_{a} \rho_{c} \sigma }}{{\mu_{c}^{2} }}} \right)^{\begin{subarray}{l} 0.29 \\ \end{subarray} } \left( {\frac{{\rho_{c} \sigma^{4} }}{{\psi \mu_{c} }}} \right)^{ - 0.13} \hfill \\ \quad \quad \quad \quad \; \times \left( {\frac{\Delta \rho }{{\rho_{c} }}} \right)^{2.86} \left( {\frac{{\mu_{d} }}{{\mu_{c} }}} \right)^{0.085} \left( {\frac{h}{{d_{a} }}} \right)^{ - 0.734} \left( {1 + L} \right)^{ - 0.34} \hfill \\ \end{gathered}$$	^26^
PDDC	Dilute sulfuric acid (4 g/L) water	$$\begin{gathered} \frac{{d_{32} }}{{\sqrt {\frac{\sigma }{\Delta \rho g}} }} = 31.7\varepsilon^{1.6} \left( {\frac{{\mu_{c} \nu_{c} }}{\sigma }} \right)^{0.116} \left( {\frac{{\nu_{d} }}{{\nu_{c} }}} \right)^{0.118} \hfill \\ \quad \quad \quad \quad \;\; \times \left[ {0.324 + \exp \left( { - 6.27\frac{Af}{{\varepsilon \left( {\frac{{g\sigma_{ * } }}{{\rho_{ * } }}} \right)^{0.25} }}} \right)} \right] \hfill \\ \end{gathered}$$	^27^
PDDC	3N nitric acid solution 30% TBP in dodecane	$$\begin{gathered} \frac{{d_{32} }}{{\sqrt {\frac{\sigma }{\Delta \rho g}} }} = 14\varepsilon^{0.3} \left( {\frac{{h_{d} }}{{2\sqrt {\frac{{\sigma_{ * } }}{{g\rho_{ * } }}} }}} \right)^{0.18} \left( {\frac{{\mu_{c} V_{c} }}{\sigma }} \right)^{0.126} \left( {\frac{{V_{d} }}{{V_{c} }}} \right)^{0.118} \left( {\frac{\sigma }{{\sigma_{ * } }}} \right)^{0.06} \hfill \\ \quad \quad \quad \quad \;\; \times \left[ {0.077 + \exp \left( { - 3.85\frac{Af}{{\varepsilon \left( {\frac{{g\sigma_{ * } }}{{\rho_{ * } }}} \right)^{0.25} }}} \right)} \right] \hfill \\ \end{gathered}$$	^28^

Angelov et al.^14^ obtained a relation based on computational fluid dynamics (CFD) for pulsed disc and doughnut columns when a drop breaks into two drops.

5 $$ We_{c} = 0.26 = \frac{{\rho_{c} kd_{\max } }}{\sigma } $$

k is a constant depends on pulse intensity.

Tenova columns have been the subject of limited studies in the literature. Among the first studies reported by Li et al.^29^, the hydrodynamic performance of a Tenova pulsed column with a standard disc-doughnut pulsed column with a length of 200 cm and a diameter of 7.6 cm for copper extraction using LIX 84 compared. As a result, in the same operating conditions, less dispersed phase holdup was observed in Tenova column compared to disc and doughnut. At low pulsation intensity, the Sauter mean drop diameter was observed to be larger in the Tenova column compared to the disc-doughnut column. In another study by Li et al.^12^, the hydrodynamic performance of a Tenova column with a standard disc-doughnut pulsed column for the 336-Shellsol 2046 system was compared. Dispersed phase holdup and Sauter mean drop diameter under different pulsation intensities and different flow rates of phases. Their results showed that compared to a standard disc-doughnut pulsed column, the drops are larger and the dispersed phase holdup is lower.

## Materials and methods

### Liquid–liquid system

The liquid–liquid system used in this study is the standard chemical system recommended by the European Federation of Chemical Engineering (E.F.C.E.). Toluene-water (high interfacial tension), isobutanol-water (low interfacial tension), and isobutyl acetate-water (medium interfacial tension) were used.

The physical properties of these systems, including the viscosity and density of each phase and surface tension, are shown in Table 2. Water saturated with solvent was used as the continuous phase and solvent saturated with water was used as the dispersed phase. Viscosity and surface tension were measured using an Ostwald viscometer (schott-Gerate GmbH, Germany), and a digital tensiometer (KRUSSN, Germany, model K10ST), respectively.

**Table 2:** Physical characteristics of the systems used at 22 °C.

Physical characteristic	Symbol	Unit	System		
			Water & isobutanol	Water & isobutyl acetate	Water & toluene
Density of continuous phase	ρ_c_	kg/m^3^	985	997	998.2
Density of dispersed phase	ρ_d_	kg/m^3^	836	870	865.2
Viscosity of continuous phase	μ_c_	m Pa s	1.51	1.03	0.963
Viscosity of dispersed phase	μ_d_	m Pa s	3. 65	0.72	0.584
Surface tension	σ	mN/m	1.75	14.1	36

### Equipment and experimental procedure

**Figure 1:**
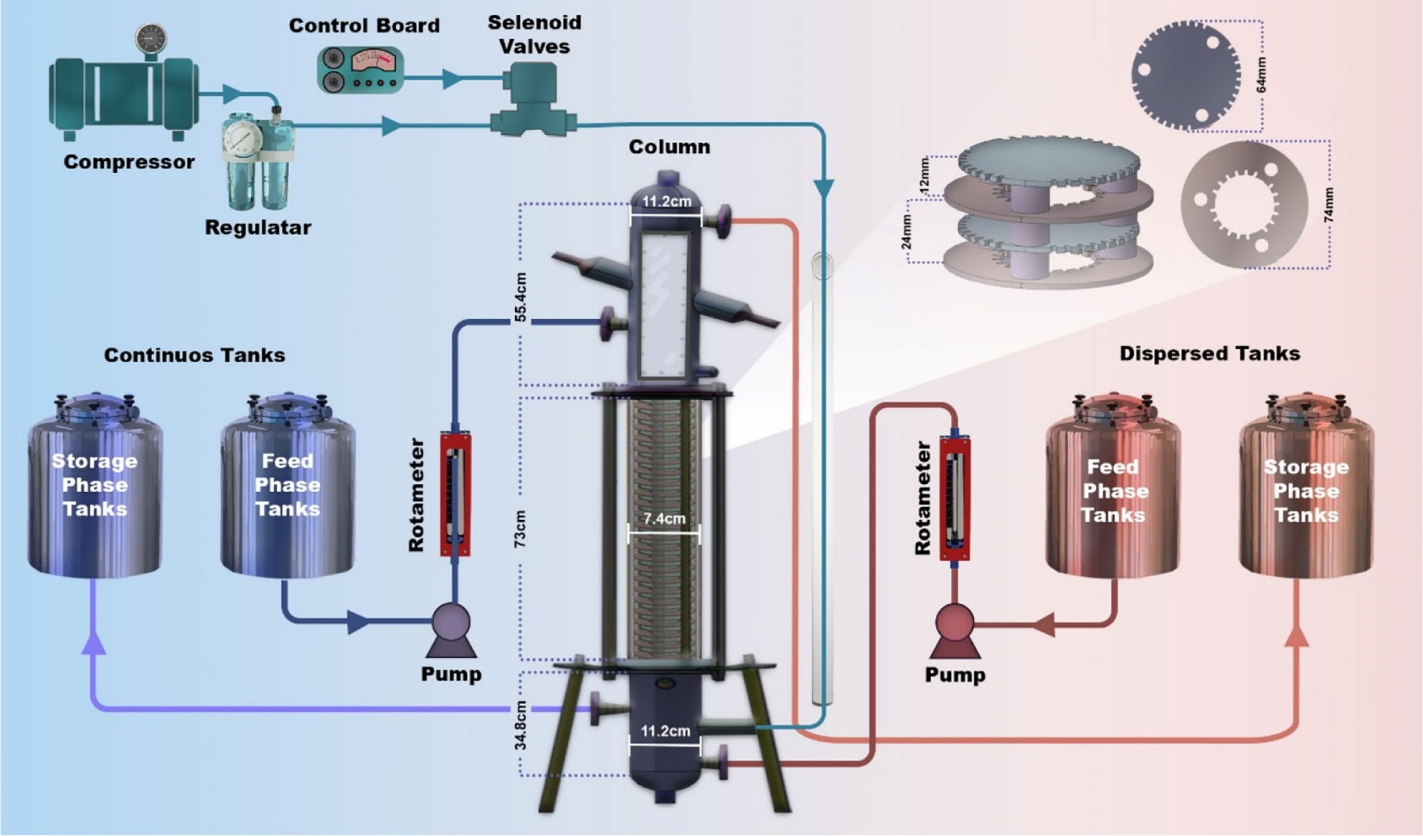
Schematic of the set-up used in this study, Tenova pulsed column and internal equipment of the column.

A schematic of the set-up used in this study is shown in Fig. 1. The active part was a tubular column with a diameter and length of 7.4 and 73 cm, which is made of Pyrex material. Its internal equipment consisted of 30 pairs of modified discs and doughnuts made of 0.2 cm thick stainless steel sheets which were placed alternately and at a distance of 1.2 cm from each other by means of Teflon spacers and were held by three support rods. The diameter of the discs, doughnuts and the holes of the doughnuts is 6.4, 7.4 and 3.4 cm, respectively and the free area fraction was 21.1%. Two precipitators were located at the top and bottom of the column with a diameter of 11.2 cm to completely separate the phases before exiting the column.

**Figure 2:**
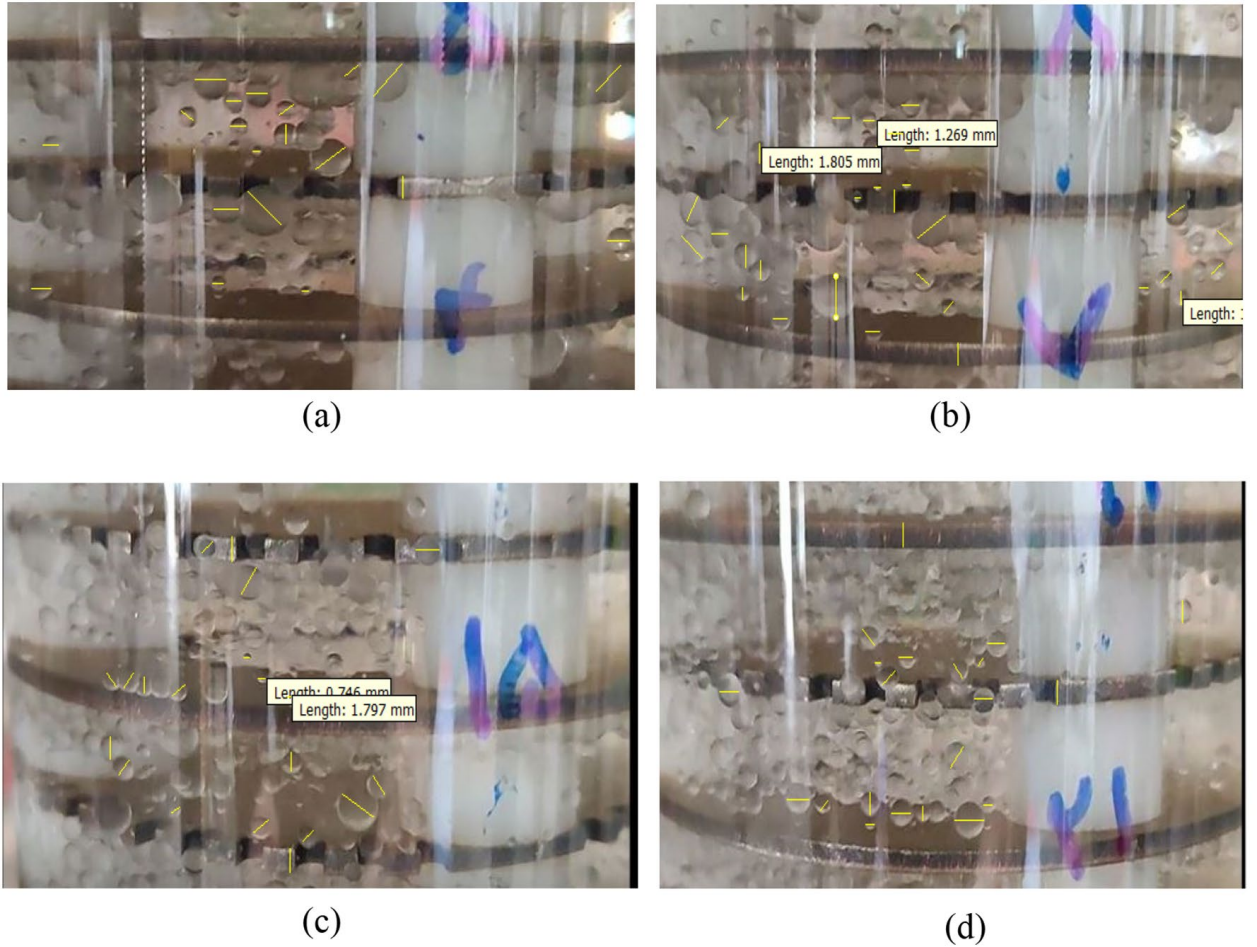
Real images of the column to check the drop size along the column related to various stages, (**a**) 5th stage, (**b**) 7th stage, (**c**) 15th stage, (**d**) 20th stage.

The drop sizes in this study were measured by photographing different parts of the column using a D3500 Nikon digital camera. The method of photographing the column was that after setting up the device in each test and setting the flow rate of the dispersed and continuous phase in the column, first the pulsation intensity was adjusted by the board controller and regulator, and the flow inlet valves were opened and then the pulse switch in the system turned on. After setting up the system, the aqueous and organic phases were allowed to flow in the column and about three volumes of the column, the flow of phases passed along the length of the column; In this case, the size of the drops remains constant and the drops reach their real size. At this time, when there is a pulse in the system, it was started to take pictures of different parts of the column. Photographs were taken from each stage separately along the length of the column and the photographs were used to calculate the diameter of the drops that were not spherical using Digimzer software. For elliptical drops both the minor and major axes, d_1_ and d_2_ were measured and the equivalent diameter, de, was calculated from Eq. 6, and an average of 200–300 drops were examined in each experiment and in total more than 10,000 drops have been analyzed. To examine the droplets, specific stages from the lower, middle, and upper segments of the column were individually selected under various test conditions. The software was employed to analyze the images captured from each stage. Calibration of the software relied on the thickness of discs and donuts as criteria, measuring all observed droplets in the images. Figure 2 provides actual images of the column as an example, showcasing analyzed images from stages 5, 7, 15, and 20, along with the respective count of analyzed droplets

6 $$ d_{e} = \left( {d_{1}^{2} d_{2} } \right)^{1/3} $$

The physical characteristics of the systems used at a temperature of 22 °C are shown in Table 2.

## Results and discussions

### The effect of operational parameters on the Sauter mean drop diameter

**Figure 3:**
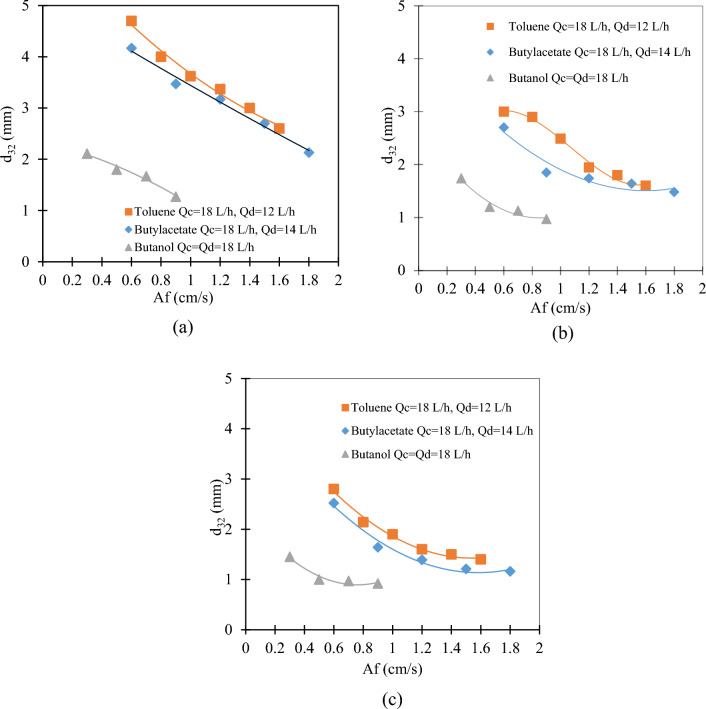
The effect of pulsation intensity on the Sauter mean drop diameter for all three systems at (**a**) stage 2; (**b**) stage 10, and (**c**) stage 15.

For all three systems at stage 2, the Sauter mean drop diameter decreases with increased pulsation intensity (see Fig. 3a). Similar results were obtained by the literature in this type of extraction column^29^.

It is expected that with the increase of the pulsation intensity, the Sauter mean drop diameter will reach its final limit, and the pulsation intensity at that point can be named the critical pulsation intensity, which can be the ultimate limit of breakage.

For all three systems, a decreasing trend is observed with increasing pulsation intensity in stages 2, 10 and 15 (Fig. 3a, b and c respectively). Comparing these three stages together shows that this reduction process starts from an average diameter of stage 10 less than stage 2 and in stage 15 an average diameter less than stage 10 and will reach the same final level at the point of critical impact intensity.

**Figure 4:**
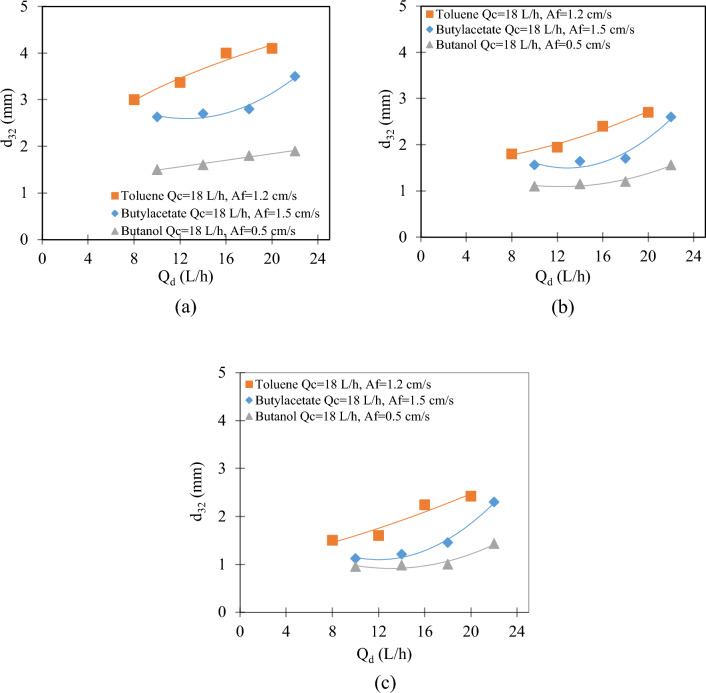
The effect of dispersed phase flow rate on the Sauter mean drop diameter for all three systems at (**a**) stage 2; (**b**) stage 10, and (**c**) stage 15.

With the increase in the flow rate of the dispersed phase in stages 2, 10, and 15, as seen in Fig. 4, the Sauter mean drop diameter increases. It seems that with the increase in the flow rate of the dispersed phase, the average diameter produced in the distributor is larger until reaching the jet point in the distributor, so the increasing trend of the average diameter of the dispersed phase seems quite logical. It shows an increasing trend for three systems, from isobutanol to toluene, due to the increase in interphase tension. Similar results were obtained in the literature in disc and doughnut impact extraction columns^26^.

The decreasing trend of these changes from stage 2 to 15 for all three systems indicates that there is a breakage along the length of the column, so the decreasing trend of the Sauter mean drop diameter along the length of the column (from stage 2 to 15) seems quite logical and this trend has been seen in all three systems.

**Figure 5:**
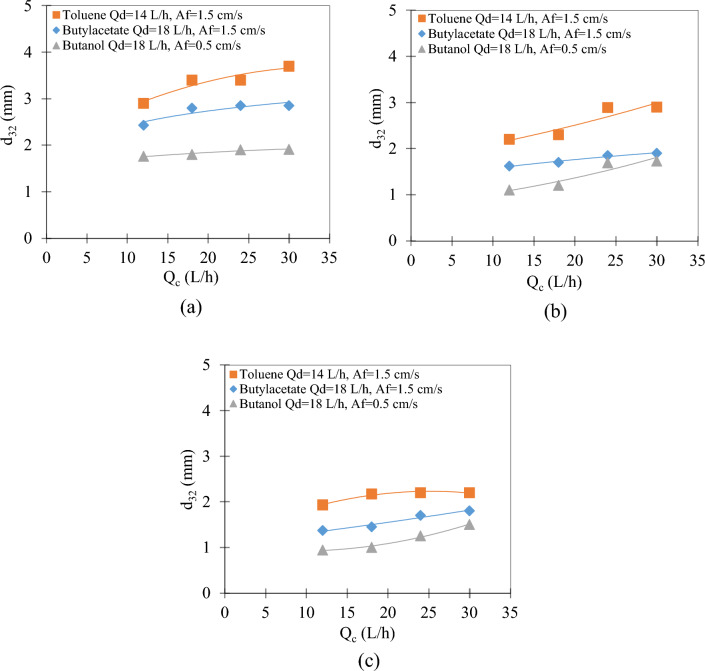
The effect of continuous phase flow rate on the Sauter mean drop diameter for all three systems at (**a**) stage 2; (**b**) stage 10, and (**c**) stage 15.

As can be seen in Fig. 5, the effect of continuous phase flow rate on the Sauter mean drop diameter is very small, but its increasing trend indicates the possibility of drop coalescence in different positions.

The decreasing trend of the Sauter mean drop diameter is in terms of the breaking of the drops along the length of the column, which indicates that the pulsation intensity was higher than the critical point ($Af_{cr}$) and the breaking along the length of the column leads to a decrease in the Sauter mean drop diameter is from stage 2 to stage 15 for all three systems.

### Drop size distribution

**Figure 6:**
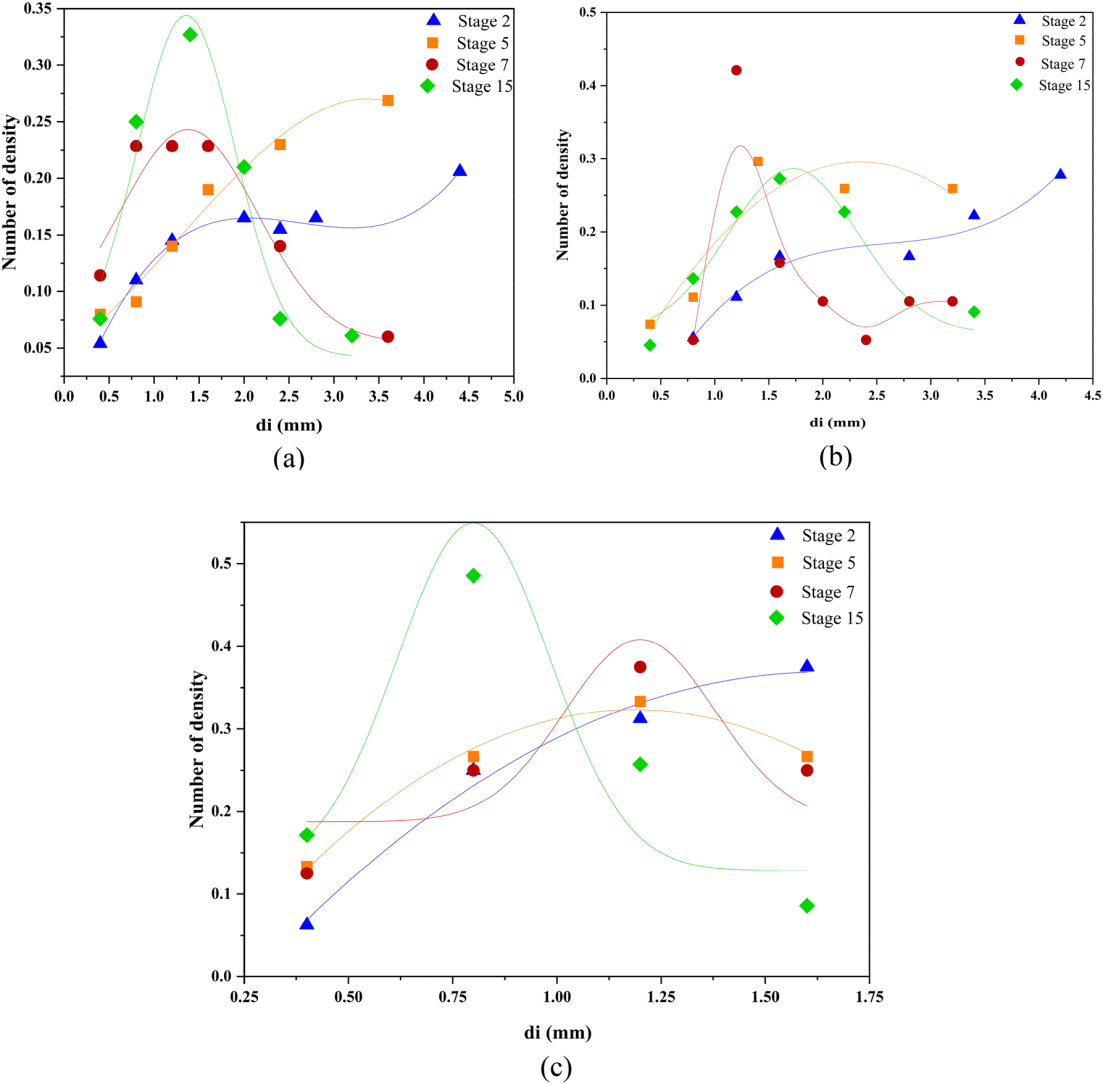
Drop size distribution for (**a**) the toluene-water system in Af = 1cm/s, Q_c_ = 18L/h, Q_d_ = 12L/h; (**b**) the isobutyl acetate-water system in Af = 1cm/s, Q_c_ = 18L/h, Q_d_ = 14L/h; (**c**) the isobutanol-water system in Af = 1cm/s, Q_c_ = Q_d_ = 18L/h.

The drop size distribution for the toluene-water system and for stages 2–7 is shown in Fig. 6a. It is evident that the breakup process occurs during the column operation and leads to a tendency towards a normal distribution.

As can be seen, the number of drops with a larger size in stage 2 is much more than the drops with a smaller size, which indicates that the process of breaking is underway, and from stages 2 to 7, the distribution tends toward normal due to the breaking of the drops. It can be seen that the distribution will be normal from stage 7 onwards.

This trend can be observed for the isobutyl acetate system as well (Fig. 6b), but as expected, the drop diameter is smaller than that of toluene. The trend mentioned for the isobutanol-water system can also be seen in Fig. 6c. It appears that along the length of the column, droplet breakage contributes to a normal distribution, predominantly observed in the middle stages. Following these middle stages, a consistent normal distribution is maintained due to a balance between breakage and coalescence, resulting in stability beyond this point.

### The correlation of predicting the Sauter mean drop diameter

To achieve the design of such columns, we need correlation to predict the Sauter mean drop diameter.

**Figure 7:**
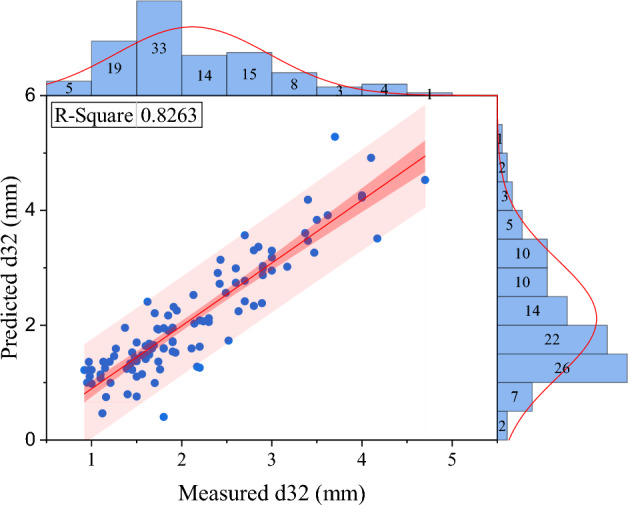
Comparing the Sauter mean drop diameter experimentally with the predicted value in the linear regression model.

**Figure 8:**
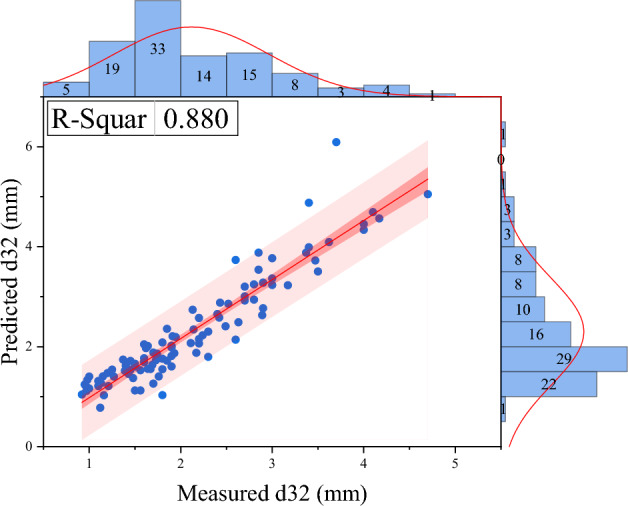
Comparing the Sauter mean drop diameter experimentally with the predicted value in the non-linear regression model.

As mentioned before, no correlation has been provided to predict this parameter specific to the Tenova column. In this study, a correlation based on operational parameters and physical characteristics of the system specific to this column is provided. In addition to predicting the Sauter mean drop diameter, changes in column length can be predicted. According to the background of the research and the identification of variables that affect Sauter mean drop diameter, using dimensional analysis, 9 products without independent dimensions were obtained to predict Sauter mean drop diameter. In order to create a linear equation between independent variables without dimension and d_32_, linear multivariate regression was used (see Figs. 7 and 8), and its equation is as follows:

7 $$ \varphi = \left( {\overbrace {{\frac{{Q_{d} }}{{Q_{c} }}}}^{{\pi_{1} }},\overbrace {{\frac{{\mu_{c} }}{{\mu_{d} }}}}^{{\pi_{2} }},\overbrace {{\frac{{\rho_{c} }}{{\rho_{d} }}}}^{{\pi_{3} }},\overbrace {{\frac{\Delta \rho }{{\rho_{d} }}}}^{{\pi_{4} }},\underbrace {{Af \times \rho_{d}^{2} \times \frac{{Q_{c} }}{{\mu_{d}^{2} }}}}_{{\pi_{5} }},\underbrace {{\sigma \times \rho_{d}^{2} \times \frac{{Q_{c} }}{{\mu_{d}^{3} }}}}_{{\pi_{6} }},\underbrace {{g \times Q_{c}^{3} \times \frac{{\rho_{d}^{5} }}{{\mu_{d}^{5} }}}}_{{\pi_{7} }},\underbrace {NS}_{{\pi_{8} }},\overbrace {{d_{32} \times \frac{{\mu_{d} }}{{Q_{c} \rho_{d} }}}}^{{\pi_{9} }}} \right) = 0 $$

8 $$ \begin{gathered} d_{32} \frac{{\mu_{d} }}{{\rho_{d} Qc}} = C_{1} + C_{2} \left( {\frac{{Q_{d} }}{Qc}} \right) + C_{3} \left( {\frac{{\mu_{c} }}{{\mu_{d} }}} \right) + C_{4} \left( {\frac{{\rho_{c} }}{{\rho_{d} }}} \right) + C_{5} \left( {\frac{{Af\rho_{d}^{2} Qc}}{{\mu_{d}^{2} }}} \right) \hfill \\ \quad \quad \quad \quad \quad + C_{6} \left( {\frac{{\sigma \rho_{d}^{2} Qc}}{{\mu_{d}^{3} }}} \right) + C_{7} \left( {\frac{{gQc^{3} \rho_{d}^{5} }}{{\mu_{d}^{5} }}} \right) + C_{8} NS \hfill \\ \end{gathered} $$

9 $$ \begin{gathered} d_{32} \frac{{\mu_{d} }}{{\rho_{d} Qc}} = {\text{C}}_{1} + {\text{C}}_{2} {\text{lg}}_{{{10}}} {(}\pi_{1} {)} + {\text{C}}_{3} {\text{lg}}_{{{10}}} {(}\pi_{2} {)} + {\text{C}}_{4} {\text{lg}}_{{{10}}} {(}\pi_{3} {)} + {\text{C}}_{5} {\text{lg}}_{{{10}}} {(}\pi_{4} {)} \hfill \\ \quad \quad \quad \quad \quad \, + {\text{C}}_{6} {\text{lg}}_{{{10}}} {(}\pi_{5} {)} + {\text{C}}_{7} {\text{lg}}_{{{10}}} {(}\pi_{6} {)} + {\text{C}}_{8} {\text{lg}}_{{{10}}} {(}\pi_{7} {)} + {\text{C}}_{9} {\text{lg}}_{{{10}}} {(}\pi_{8} {)} + {\text{C}}_{10} \pi_{3} \pi_{5} \hfill \\ \end{gathered} $$

The constants of linear and non-linear regression are given in Table 3.

**Table 3:** Constants of linear and non-linear regression.

Correlation		Linear	Non-linear
Constants	C_1_	6.44E−04	2237.479
	C_2_	3.98E−04	4.95 E−04
	C_3_	− 6.06E−04	− 362.73
	C_4_	4.56E−04	− 15,582.159
	C_5_	− 1.895E−09	1507.563
	C_6_	8.24E−10	− 1.13 E−03
	C_7_	2.239E−21	123.883
	C_8_	− 2.279E−5	− 41.294
	C_9_	–	− 3.26 E−04
	C_10_	–	2.398 E−09
	AARD%	16.67	14.8

In this study, the gene expression programming (GEP) model was used to predict d_32_ with high accuracy and also to validate the model with higher reliability.

The main advantage of this method is that, unlike other artificial intelligence methods, it provides the user with an equation. To model d_32_ using this technique, the parameters of the model should be set first and then the model should be developed.

Here, 4 genes have been obtained, these genes must be connected to each other to create an equation. Therefore, the main equation used to predict d_32_ is as follows:

10 $$ \begin{gathered} Gen1 = \left[ {\sin (\tan (\pi_{3} \pi_{4} ))\pi_{1} \sin (6.57)} \right]^{3} \hfill \\ Gen2 = \left[ {(\pi_{3} - \pi_{2} + \frac{1}{{\pi_{2} }})\pi_{8} \pi_{5} \pi_{4} \pi_{3} } \right]^{ - 1} \hfill \\ Gen3 = \frac{1}{{\pi_{7} - x_{6} }}(\pi_{8} - \pi_{5} )(\pi_{2} + \pi_{8} )(2\pi_{5} ) \hfill \\ Gen4 = e^{{[(\pi_{2} + 6.63)(\pi_{4} - \pi_{3} ) - \cos (\pi_{2} )\pi_{4} ]}} \hfill \\ d_{32} \times \frac{{\mu_{d} }}{{Q_{c} \rho_{d} }} = Gen1 + Gen2 + Gen3 + Gen4 \hfill \\ \end{gathered} $$

**Figure 9:**
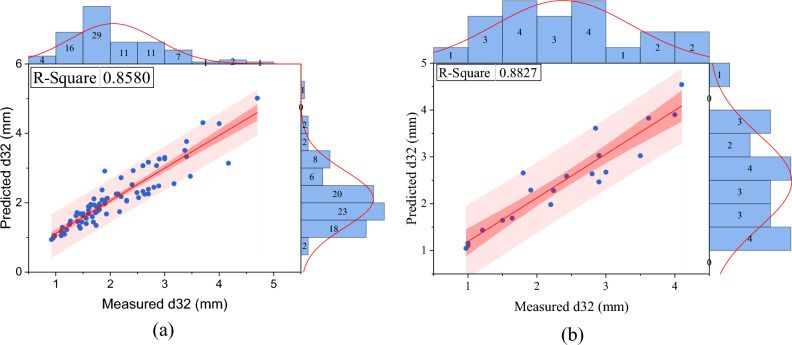
Predicted and measured values of d_32_ in the (**a**) training and (**b**) testing section of the GEP model.

A comparison of the predicted and measured values using the GEP model is shown in Fig. 9. These results show that the value of d_32_ is predicted with very high accuracy and the validation of the model is done with high confidence.

**Figure 10:**
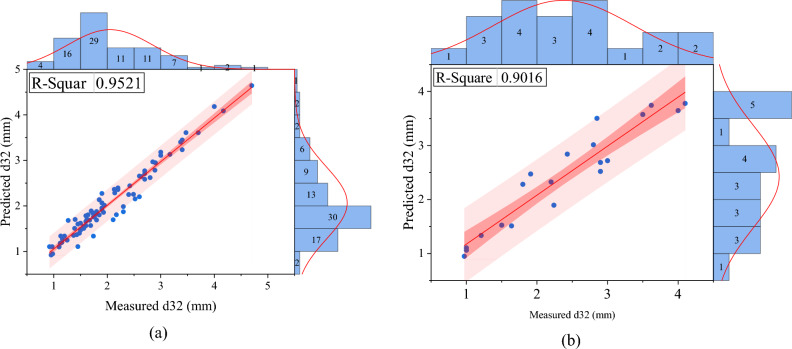
Predicted and measured values of d32 in the (**a**) training and (**b**) testing section of the MLP neural network model.

In order to compare the developed model with other artificial intelligence methods, a multilayer perceptron (MLP) artificial neural network was used as a powerful artificial intelligence method. This method has the ability to predict at a high level of accuracy. The dimensionless variables were called model inputs to the neural network and the value of d_32_ was predicted. The modeling results are shown in Fig. 10.

Two correlation for predicting the Sauter mean drop diameter reported in the literature^27,28^ were evaluated for the experimental data of the present study. The average absolute relative deviation (AARD) was reported.

11 $$ AARD = \frac{1}{n}\mathop \sum \limits_{1}^{n} \frac{{\left| {predicted\;value - experimental\;value} \right|}}{experimental\;value}*100\% $$

**Figure 11:**
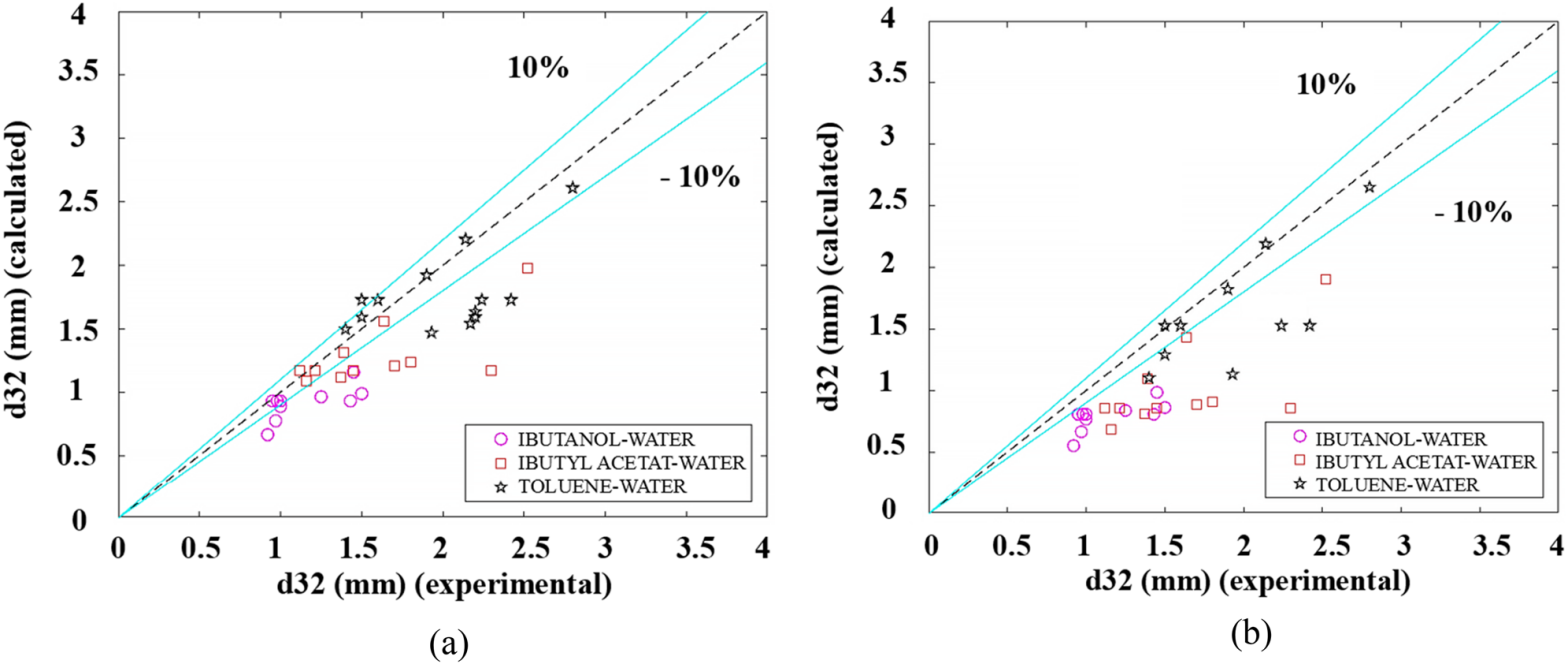
Comparing the Sauter mean drop diameter experimentally with the predicted value; (**a**) Wang et al. (**b**) Sarkar et al.

Figure 11 compares the Sauter mean drop diameter measured experimentally with the predicted value by the mentioned correlations.

In Table 4, the comparison of previously reported correlations for predicting the Sauter mean drop diameter and the present work.

**Table 4:** AARD for predicting the Sauter mean drop diameter based on the previous and the present work.

AARD (%)	References
17.1	Wang et al.
29	Sarkar et al.
16.67	Present work MLR
14.8	Present work MNLR
9	Present work ANN
11.3	Present work GEP

### Sensitivity analysis

Among the useful measures after modeling is determining the sensitivity of the target parameter to the input parameters. Typically, to determine the sensitivity value of the input parameters on the target, by removing one of the input parameters, the changes in the error value of the test data are checked. The severity of the increase in error indicates the greater impact of the omitted parameter on the results. In this study, after applying the sensitivity using the Cosine Amplitude Method (CAM), the intensity and impact of the input parameters on the output was calculated. This method was first developed by Yang and Zhang^30^ and then by Jong and Lee^31^ and used to determine the similarity of correlations between related parameters. In this method, an m-dimensional space where m is the number of input parameters is assumed:

12 $$ X = \left\{ {x_{1} ,x_{2} ,x_{3} , \ldots ,x_{m} } \right\} $$

Each member of the input parameters such as X is connected to the objective function by a length vector:

13 $$ X_{i} = \left\{ {x_{i1} ,x_{i2} ,x_{i3} , \ldots ,x_{im} } \right\}_{1} $$

**Figure 12:**
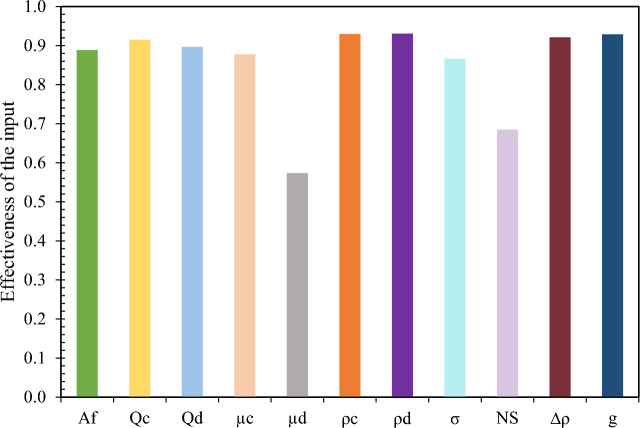
Value of r_ij_ factor in d_32_ sensitivity analysis.

Therefore, each data point is a point in the m-dimensional space that requires m coordinate components for a complete description. The strength of the correlation (r) between both variables can be calculated as follows:

14 $$ r_{ij} = \frac{{\sum\limits_{k = 1}^{m} {x_{ik} \cdot x_{jk} } }}{{\sqrt {\left( {\sum\limits_{k = 1}^{m} {x_{ik}^{2} } } \right) \cdot \left( {\sum\limits_{k = 1}^{m} {x_{jk}^{2} } } \right)} }} $$

where x_i_ and x_j_ are independent and dependent variables, respectively. In this way, the impact of each of the input parameters is calculated. The greater the impact of the input parameter on the target (output), the r_ij_ approaches 1, and on the other hand, if there is no impact of the input parameter on the target output, the value of r_ij_ tends to zero. Normally, the value of r_ij_ > 0.9 indicates a significant effect of the independent parameter on the output, and values < 0.8 indicate its weak effect on the output variable. Regarding the data used in this study, the sensitivity level of each of the input parameters is shown in Fig. 12. The results show that all independent parameters have an effect on d_32_ changes.

## Conclusions

The Tenova column, which has been introduced in recent years, has been built and tested on a pilot scale. Equations (8), (9), (10) were proposed for predicting of mean drop size along the column using the dimensional analysis approach. The proposed correlations are compared with the correlation presented in the literature. The results obtained from this work in the middle stages where the distribution of drops is completely normalized are in good agreement with the results of other researchers (error less than 29%) and an error of less than 12% has been obtained by editing the correlation presented with the GEP method. The correlation presented based on the stage number can be used in the length of the column, which is strongly recommended for the design of such columns instead of the average diameter. In addition to the average diameter and its changes along the length of the column, the distribution of drops and how it changes along the length of the column has been obtained experimentally for all the mentioned systems. The results are justifiable. For each distribution curve, at least 200–300 drops and in total, more than 10,000 drops have been analyzed in different operating conditions in this research work.

## Data Availability

The datasets used and/or analyzed during the current study available from the corresponding author on reasonable request.

## List of symbols

A: Amplitude of pulsation (m)
C_o_: Orifice coefficient (-)
d: Drop diameter (m)
b: Deceleration, m/s*
V_m_: Velocity (m/s)
C_D_: Drag coefficient
d_a_: Doughnut aperture diameter (m)
d_32_: Sauter Mean drop diameter (m)
d_e_: Equivalent diameter (m)
d_i_: Diameter (m)
d_o_: Plate perforation diameter (m)
f: Frequency of pulsation (1/s)
g: Acceleration due to gravity (m/s)
h: Disc to doughnut spacing (m)
h_c_: Compartment height (m)
h_d_: Disc spacing (m)
h_p_: Plate spacing (m)
NS: Stage number
Q_c_: Continuous phase volume floe rate (m^3^/s)
Q_d_: Dispersed phase volumetric flow rate (m^3^/s)
R: Flow ratio
Ψ: Mechanical power dissipation per unit mass, W/kg
ρ_*_: Reference dynamic viscosity of water at 298 K [0.001 kg/(m s)]
σ_*_: Reference surface tension of water at 298 K [0.0728 N/m] Greek symbols
σ: Interfacial tension (N/m)
Δρ: Density difference between phases (kg/m^3^)
*μ*: Viscosity (Pa s)
*ρ*: Density (kg/m^3^)
K: Viscosity ratio (-)
*ε*: Fractional free area (-)
Ψ: Power dissipated per unit mass (m^2^/s^3^) subscripts
We: Weber number, dimensionless

## Notation

c: Continuous phase
d: Dispersed phase

## References

[1] Rydberg J (2004). Solvent Extraction Principles and Practice, Revised and Expanded.

[2] Leonard RA (1988). Recent advances in centrifugal contactor design. Sep. Sci. Technol..

[3] Zhao M, Cao S, Duan W (2014). Effects of some parameters on mass-transfer efficiency of a ϕ20 mm annular centrifugal contactor for nuclear solvent extraction processes. Prog. Nucl. Energy.

[4] Sharma JN, Kumar A, Kumar V, Pahan S, Janardanan C, Tessi V, Wattal PK (2014). Process development for separation of cesium from acidic nuclear waste solution using 1,3-dioctyloxycalix [4] arene-crown-6 + isodecyl alcohol/ n -dodecane solvent. Sep. Purif. Technol..

[5] Li Z, Zhao H, He S, Chen M, Liu C, Li R, Zhang L, Li Q (2016). A systematic research on solvent extraction process for extracting 233U from irradiated thorium. Hydrometallurgy.

[6] Dartiguelongue A, Chagnes A, Provost E, Fürst W, Cote G (2016). Modelling of uranium(VI) extraction by D2EHPA/TOPO from phosphoric acid within a wide range of concentrations. Hydrometallurgy.

[7] Quinn JE, Soldenhoff KH (2015). Process for uranium recovery using Cyanex 272. Hydrometallurgy.

[8] Smirnov AL, Skripchenko SY, Rychkov VN, Pastukhov AM, Shtutsa MG (2013). Uranium stripping from tri-n-butyl phosphate by hydrogen peroxide solutions. Hydrometallurgy.

[9] Ferreira AE, Agarwal S, Machado RM, Gameiro MLF, Santos SMC, Reis MTA, Ismael MRC, Correia MJN, Carvalho JMR (2010). Extraction of copper from acidic leach solution with Acorga M5640 using a pulsed sieve plate column. Hydrometallurgy.

[10] Gameiro MLF, Machado RM, Ismael MRC, Reis MTA, Carvalho JMR (2010). Copper extraction from ammoniacal medium in a pulsed sieve-plate column with LIX 84-I. J. Hazard. Mater..

[11] Schön J, Schmieder H, Kanellakopulos B (1990). Operating experience with Minka: U-Pu separation in the presence of technetium with an organic continuous pulsed column. Sep. Sci. Technol..

[12] Li W, Wang Y, Mumford KA, Smith KH, Stevens GW (2018). Prediction of holdup and drop size distribution in a disc-doughnut pulsed column with tenova kinetics internals for the water-Alamine 336 system. Hydrometallurgy.

[13] Chaturabul S, Wannachod P, Rojanasiraprapa B, Summakasipong S, Lothongkum AW, Pancharoen U (2012). Arsenic removal from natural gas condensate using a pulsed sieve plate column and mass transfer efficiency. Sep. Sci. Technol..

[14] Angelov, G., Gourdon, C., LineÂ, A. Solvent extraction in the process industries. In *Proceedings of ISEC'93* 1183–1190 (1993).

[15] Kolmogorov, A. N. Berichte der Akademie der Wissenschaftender USSR **466**(5), 815–830 (1949). (German translation in Sammelband zur statistischen Theorie der Turbulenz, Akademie Verlag 151–56 (1958)).

[16] Kolmogorov, A. N. Berichte der Akademie der Wissenschaftender USSR, **430**(4), 199–305 (1949). (German translation in Sammelband zur statistischen Theorie der Turbulenz, Akademie Verlag 71–76 (1958)).

[17] Hinze JO (1955). Fundamentals of the hydrodynamic mechanism of splitting in dispersion processes. AIChE J..

[18] Shinnar R, Church JM (1960). Statistical theories of turbulence in predicting particle size in agitated dispersions. Ind. Eng. Chem..

[19] Jealous AC, Johnson HF (1955). Power requirements for pulse generation in pulse columns. Ind. Eng. Chem..

[20] Haverland, H. Untersuchung zur tropfendispergierung in flüssigkeitspulsierten siebboden-extraktionskolonnen. Dissertation technische universität clausthal (1988).

[21] Pietzsch W, Pilhofer TH (1984). Calculation of the drop size in pulsed sieve-plate extraction columns. Chem. Eng. Sci..

[22] Kagan SZ (1965). Some hydrodynamic and mass-transfer problems in pulsed sieve-plate extractors. Int. Chem. Eng..

[23] Sreenivasulu K, Venkatanarasaiah D, Varma YBG (1997). Drop size distributions in liquid pulsed columns. Bioprocess Eng..

[24] Kumar A, Hartland S (1996). Unified correlation for the prediction of drop size in liquid−liquid extraction columns. Ind. Eng. Chem. Res..

[25] Van Delden ML, Kuipers NJM, de Haan AB (2006). Extraction of caprolactam with toluene in a pulsed disc and doughnut column—part I: Recommendation of a model for hydraulic characteristics. Solvent Extr. Ion Exch..

[26] Torab-Mostaedi M, Ghaemi A, Asadollahzadeh M (2011). Flooding and drop size in a pulsed disc and doughnut extraction column. Chem. Eng. Res. Des..

[27] Wang Y, Smith KH, Mumford KA, Yi H, Wang L, Stevens GW (2016). Prediction of drop size in a pulsed and non-pulsed disc and doughnut solvent extraction column. Chem. Eng. Res. Des..

[28] Sarkar S, Sen N, Singh KK, Mukhopadhyay S, Shenoy KT (2019). Liquid-liquid dispersion in pulsed disc and doughnut column and pulsed sieve plate column: A comparative study. Prog. Nucl. Energy.

[29] Li W, Wang Y, Mumford KA, Smith KH, Stevens GW (2017). Comparison of the hydrodynamic performance of pulsed solvent extraction columns with Tenova pulsed column kinetics internals and standard disc and doughnut internals for copper extraction using the LIX 84 system. Solvent Extr. Ion Exch..

[30] Yang Y, Zhang Q (1997). A hierarchical analysis for rock engineering using artificial neural networks. Rock Mech. Rock Eng..

[31] Jong Y-H, Lee C-I (2004). Influence of geological conditions on the powder factor for tunnel blasting. Int. J. Rock Mech. Min. Sci..

